# Apoyando a la juventud [supporting the youth]: Latinx caregivers’ assessment of youth mental health service need and utilization on the Caregiver Support Services Questionnaire

**DOI:** 10.1371/journal.pmen.0000345

**Published:** 2025-06-20

**Authors:** Alejandro L. Vázquez, Tyson S. Barrett, Amanda Venta, María de la Caridad Alvarez, Melanie M. Domenech Rodríguez

**Affiliations:** 1 Department of Psychology, University of Tennessee - Knoxville, Knoxville, Tennessee, United States of America; 2 Highmark Health, Pittsburgh, Pennsylvania, United States of America; 3 Department of Psychology, University of Houston, Houston, Texas, United States of America; 4 Department of Psychology, Utah State University, Logan, Utah, United States of America; Universidad de Huelva Facultad de Educacion, SPAIN

## Abstract

Established service utilization measures depend on labor- and time-intensive interviews that limit the feasibility of large-scale studies seeking to understand utilization patterns of youth mental health services (MHS) among Latinx families. The present study describes the development of the Caregiver Support Services Questionnaire (CSSQ), a self-administered measure of perceived need for and utilization of common youth MHS. Data from 598 Latinx caregivers of youths ages 6–18 on the CSSQ were examined to (a) determine whether the CSSQ can be represented by cumulative scores and individual indicators and (b) establish the construct validity of the CSSQ. Correlations between CSSQ items had small to moderate effect sizes, suggesting that item responses within each scale were interrelated but provided unique information. Confirmatory factor analyses indicated that unidimensional factors for the perceived need and service utilization scales fit the data well and have excellent internal consistency. The severity of youth emotional and behavioral problems was generally associated with increased odds of reporting support service need and utilization on the CSSQ, which provides support for the construct validity of the cumulative scales and individual indicators. Findings support the use of the CSSQ as both cumulative scores and individual items to study support service need and utilization patterns associated with youth mental health problems among Latinx families.

## Introduction

Disparities in mental health services (MHS) access and quality of services are well documented among Latinx youth [1]. These disparities are gaining attention at the federal level, including the 2021–2025 strategic plan developed by the National Institute on Minority Health and Health Disparities highlighting a need to identify factors contributing to inequitable access, engagement, and quality of MHS among minoritized youths [2]. While these efforts are underway and advances have been made in our understanding of factors contributing to mental health disparities among Latinx youths (e.g., identification of perceptual and structural barriers; patterns and correlates of traditional MHS use; [3–6]), several limitations associated with established service utilization measures may reduce the feasibility of broader examinations of help-seeking patterns among these families. Established measures require labor- and time-intensive interviews, do not assess caregiver perceived need for services, and do not include modern (i.e., telepsychology) and informal sources of support that Latinx caregivers may access in response to youth mental health problems (e.g., community-led parenting classes or mentorship programs; [7–9]). The present study sought to address this measurement gap through the development of a self-administered questionnaire that could support large-scale quantitative research seeking to identify targets to reduce youth mental health disparities among Latinx families.

### Help-seeking models

MHS utilization models provide guidance on important outcomes and predictors implicated in help-seeking behaviors among caregivers. Help-seeking pathways begin with caregiver recognition of a youth problem requiring intervention through clinical assessment (e.g., symptoms, diagnosis) and/or perceived need (e.g., subjective, cultural definition of problem as a mental health concern; [10,11]). Problem recognition informs subsequent decisions to seek help and the selection of services that are perceived to be both a congruent means of intervention and consistent with interpretations regarding the cause of youths’ presenting problems [10,11].

Latinx caregivers may seek help from traditional outpatient counseling (e.g., psychological counseling, telepsychology) and crisis services (e.g., hotline, psychiatric hospitalization), and/or informal (e.g., parenting classes, mentorship programs, friends and family, online support groups), non-specialty (e.g., school counseling, pediatricians), and religious supports (e.g., ministers or faith healers) that may or may not be delivered by mental health providers [6,12–17]). While the perceived need for MHS is often associated with subsequent decisions to seek youth support services, a wide array of factors moderate this relationship including sociocultural values and beliefs regarding mental illness, the social desirability of help-seeking, and barriers to accessing formal MHS [11,18]. These conceptual models suggest that research seeking to understand help-seeking pathways among Latinx caregivers should incorporate measures of youth problem recognition (i.e., clinical and/or perceived need) and utilization of a wide variety of supports [10,11].

### MHS use and access disparities among Latinx youth

Despite the evident need for MHS among Latinxs, several barriers make it difficult for this group to access traditional MHS (e.g., long-term, individual psychotherapy). Latinx caregivers may perceive their child’s problems to be normal despite reporting that they have clinically elevated emotional and/or behavioral problems [18]. When Latinx caregivers decide to seek help, they often report not knowing how or where to access traditional MHS for youths [18]. Additional barriers to youth MHS utilization among Latinx caregivers include stigma associated with traditional mental health supports, fear associated with legal repercussions and documentation status, and concerns surrounding cultural mismatch and racism/discrimination [19,20]. These barriers to accessing traditional mental health supports may drive Latinxs to seek out alternative supports during times of great need. Latinxs who report the highest need for MHS also access the widest variety of services, utilizing professionals within the mental healthcare and general healthcare fields as well as clergy, family, and social supports [21]. Latinx caregivers also access a wide diversity of services for their children including professionals (e.g., medical doctors, school professionals), counselors, family/friends, religious supports, mentorship programs, and didactics (e.g., parenting classes; [6,22]).

Available literature regarding need and utilization patterns for these alternative service types is sparse and little is known about what predicts engagement with alternative youth service types, leaving scientists with an incomplete picture of Latinx caregivers’ perceived need for services and patterns of use. This knowledge gap is partly attributable to a lack of measurement tools (a) available to assess service need and utilization of alternative service types and (b) that capture the data in a scalable and easy-to-administer format.

### Measures of MHS utilization

Well-established youth MHS utilization measures require labor- and time-intensive interviews. The Child and Adolescent Services Assessment (CASA; [7]), the Services Assessment for Children and Adolescents (SACA; [8]), and the Services for Children and Adolescents – Parent Interview (SCA-PI; [9]) are commonly used semi-structured interviews in the MHS literature.

The CASA is the most comprehensive of the three interviews and assesses caregiver-reported utilization of 31 youth support services in the last year (e.g., psychological counseling, crisis hotlines, hospitalization, internet support groups, doctors, school professionals, social supports, religious supports) and includes two follow up questions for each service assessed (i.e., reason for use and use in the last year; [7]). However, the administration of the CASA requires an interview that can take as long as 20 minutes with high service users, which requires significant resource allocation and reduces the feasibility of administration when recruiting large samples [7]. The SACA and SCA-PI interviews are shorter in length (i.e., 5 and 6 items with multiple open-ended questions) but assess a narrower range of youth support services [8,9]. These measures generally do not assess caregiver-reported need for services, which precludes examinations of whether caregivers would have liked to utilize certain sources of support but were unable to access them. Furthermore, these measures do not include questions regarding the need/utilization of other sources of support that caregivers may associate with youth mental health problems (e.g., telepsychology services, mentorship programs, parenting classes). These limitations highlight the unique contribution to the literature made by having a self-administered measure for caregivers that can assess both the need for and utilization of common sources of support for youths.

These service utilization measures are often used as item-level outcomes or as cumulative scores [12,23,24]. Using individual items can facilitate screening efforts as the entire scale does not need to be administered to gather relevant information about service utilization. Individual items representing service use are also often used to gather descriptive information and serve as outcome variables in logistic regression models to help identify correlates associated with help-seeking among families [5,12,25]. Cumulative scores representing service need and utilization are commonly used in path analyses examining potential mechanisms underpinning youth MHS help-seeking behaviors among minoritized caregivers [26,27]. However, representing service utilization measures as cumulative scores requires that items perform adequately together in representing a continuous latent construct [27].

### Caregiver Support Services Questionnaire

The Caregiver Support Services Questionnaire (CSSQ) was created for the Pathways to Latinx Mental Health study. The Pathways to Latinx Mental Health study was a national online survey that sought to identify youth MHS help-seeking patterns and related correlates among Latinx caregivers [4]. The CSSQ is a self-administered measure intended to assess caregivers’ perceived need for and utilization of a variety of youth support services [4]. The CSSQ was created due to limitations associated with existing service utilization measures such as (a) burdensome interview-based administration, (b) lack of evaluation of perceived need, and (c) absence of questions regarding modern service types, specifically telepsychology, mentorship programs, and parenting classes [7–9].

Youth service items included in the CSSQ were informed by the literature and existing measures of youth service utilization and adapted to inquire about both need and access to modern sources of support (i.e., psychological counseling/therapy, psychiatric hospitalization, mentorship programs, school counseling, primary care physicians, parenting classes, minister or faith healer, social supports [family/friends], telepsychology; [7–9,28]). The CSSQ includes measures of two separate constructs along the help-seeking pathway (i.e., perceived need, service utilization; [10,11]). Prior research conducted as part of the Pathways to Latinx Mental Health study found that Latinxs generally reported higher rates of service need and use on all CSSQ items when their child had clinically elevated mental health problems (i.e., dichotomous indicator of internalizing or externalizing problems; [4]). However, research has yet to examine whether the CSSQ may also be represented as cumulative scales to aid the study of caregiver help-seeking behaviors in response to youth emotional and behavioral problems.

### The present study

The need for psychometric data on a measure of MHS utilization that can be more easily administered broadly is clear in the extant literature. The need for a scalable measure can be addressed via a questionnaire-based assessment of caregiver-reported need and utilization of youth support services for use in quantitative analyses of help-seeking behaviors and related correlates among Latinx families. The present study aimed to (a) determine whether the CSSQ can be represented by cumulative scores and individual indicators and (b) establish the construct validity of the CSSQ by examining whether cumulative scores and individual responses on the CSSQ are related to youth mental health problem severity (i.e., emotional and behavioral problems). We expected that the CSSQ may be represented by cumulative scores as caregivers often perceived a need for and seek help from various sources of support to meet the developmental needs of youths [6,12]. We expected caregivers to have higher cumulative scores and greater odds of endorsing individual items on the CSSQ as the severity of youth mental health problems increased [9,23].

## Materials and methods

### Ethics statement

Participants who were eligible for the study were asked to read a letter of information describing the purpose of the survey and provided consent prior to engaging in the study. Approval to conduct the present study was obtained from the Utah State University Institutional Review Board (protocol # 11045).

### Procedures

Data were from Latinx caregivers of youths between 6–18 years-old who participated in the first phase of the Pathways to Latinx Mental Health study conducted between May 21, 2020 and June 18, 2020 [4]. The Pathways to Latinx Mental Health study sought to examine youth MHS preferences, utilization patterns, and related correlates among Latinx caregivers within a nonclinical sample. Participants were recruited through a nationwide online survey panel conducted by Qualtrics ($12.50 cost per participant). Survey panels consist of individuals who have signed up to be contacted regarding opportunities to participate in online research studies.

Qualtrics distributed emails to members of the survey panel that informed them of the nature of the survey, the required time, compensation, and a link to the survey. Participants then completed screening questions to confirm their eligibility for the study. Those who met inclusion criteria were asked to read a letter of information regarding the nature of the study and provided informed consent to participate. Individuals who provided their consent then completed a 20-min survey gathering information on family demographics, need/utilization of youth support services, and youth emotional and/or behavioral problems. Participants with multiple children were asked to report on the child who presented the most challenges to them as a parent.

Survey panels yield data that is comparable in quality to traditional in-person survey administrations when validation methods are used (i.e., in-person paper and pencil; [29]). We utilized several contrasting validation methods to identify and remove participants who provided low-quality data (i.e., logical statements, directed queries, open-ended queries, response time and pattern, honesty check, and response consistency; [30]). Internet protocol (IP) addresses were also checked to confirm that participants were completing the survey within the United States (U.S.) and that participants did not complete the survey more than once. Consistent with Qualtrics’ terms of service and procedures outlined in the consent documents, Qualtrics only provided compensation to participants who completed the entire survey and demonstrated patterns associated with high-quality data as identified by validity checks [29,31]. Qualtrics provided compensation to participants in the form of points that participants could redeem for rewards.

Inclusion criteria for the Pathways to Latinx Mental Health study were (a) identifying as Latinx, (b) being a caregiver to at least one youth between the ages 6–18, (c) ability to complete the survey in English, and (d) currently residing in the U.S. Of those who attempted the survey (*n *= 3,149), a third (*n* = 1,128) met the inclusion criteria. Participants were excluded if they did not provide consent to participate (*n* = 17), provided poor-quality data as identified by attention checks (*n *= 235), or did not complete the entire survey (*n* = 278).

### Participants

The final sample consisted of 598 caregivers that were, on average, 38.53 years old (*SD* = 9.08), predominately women (70.2%, *n* = 420), biological parents (94.5%, *n* = 565), mostly first (24.2%, *n* = 145; i.e., participants born in another place, but now live in the U.S. or U.S. territory) and second generation in the U.S. (47.3%, *n* = 283; i.e., participant was born in U.S. but at least one caregiver was born abroad), and preferred speaking English and Spanish equally (45.3%; *n* = 271). Caregivers who reported being first-generation in the U.S. (*n* = 145) were most frequently born in Mexico (*n* = 37, 6.2%), Venezuela (*n* = 17, 2.8%), Dominican Republic (*n* = 18, 3%), Peru (*n* = 11, 1.8%), Cuba (*n* = 8, 1.3%), and El Salvador (*n* = 7, 1.2%). Some first-generation caregivers reported being born in the U.S. territory of Puerto Rico (*n* = 43, 7.2%). Youths were nearly 12 years old on average (*M* = 11.87; *SD* = 3.36), were balanced on binary gender (54.8% were boys; *n* = 328), and had health insurance (private 49.8%, *n* = 298; public 46.5%, *n* = 278).

### Measures

#### Demographics.

Caregivers reported on their demographic information (i.e., age, binary gender, generational status in the U.S., preferred language, educational attainment, household income) and that of their child (i.e., age, binary gender, insurance status).

#### Caregiver Support Services Questionnaire.

The CSSQ is composed of 22 items querying caregivers’ perceived need for and utilization of a variety of youth support services in the last year. Caregivers report whether their child *“needed or would have benefited from”* and *“received”* help for their youth from each service. Responses were *yes* (1) or *no* (0). The CSSQ is available in the supplemental materials for this article under S1 Text.

#### Youth mental health problems.

Caregivers reported on the frequency of their youth’s behavioral and emotional problems in the last 6 months on the Child Behavior Checklist (ages 6–18 CBCL; [32]). Responses were *not true* (0), *sometimes true* (1), or *often true* (2). CBCL items form a total composite score representing youth internalizing (e.g., depression, anxiety) and externalizing problems (e.g., aggression, rule-breaking behavior), from which a dichotomous variable can be calculated to indicate whether youth have or do not have clinically elevated problems (i.e., *T* score above 63). The CBCL had excellent internal consistency for the total problems scale (α = .98) within the present sample.

#### Analytic plan.

Tetrachoric correlations were used to examine the strength of relationships between items within the perceived need and service use scales. Confirmatory factor analysis using the diagonally weighted least squares (DWLS) estimator with tetrachoric correlations were then used to determine whether the perceived need and service utilization measures could be represented by cumulative latent scores [33]. This approach allows for the estimation of the underlying latent structure of measures composed of binary indicators [33]. Model fit was assessed with comparative fit indices and their recommended cut offs (i.e., Root Mean Square Error of Approximation [RMSEA; good < .06, acceptable < .08, poor fit > .10], Comparative Fit Index [CFI; ≥ .95 good fit], Tucker-Lewis Index [TLI; good fit ≥ .95]; [34]). Cronbach’s alpha coefficients were also calculated to examine the internal consistency of the perceived need and service utilization scales. Construct validity was established by examining associations between CSSQ cumulative and individual indicators and youth mental health problem severity using appropriate test for the variable types being examined (i.e., Kruskal-Wallis rank sum test, logistic regressions analyses, Kendall’s Tau; [9]).

## Results

Caregivers reported that 27.8% (*n* = 166) of youths had total problems within the clinical range in the last 6 months. The average severity of youth problems, as measured by the CBCL T-scores, was 53.24 (*SD* = 15.56) for the total problem scale. See Table 1 for service need and utilization indicators by total problem severity.

**Table 1:** Caregiver reported youth support service need and utilization during the last year by clinical problem status (*N* = 598).

		CBCL total scale mean by item endorsement		
	Total	Yes	No	*p*-value
	*F(%)*	*M(SD)*	*M(SD)*	
**Perceived need**				
Psychological counseling	271 (45.3%)	63.27 (11.80)	44.93 (13.23)	<.001
Crisis hotline	55 (9.2%)	70.76 (12.89)	51.47 (14.67)	<.001
Psychiatric hospitalization	42 (7%)	75.07 (10.63)	51.59 (14.60)	<.001
Mentorship programs	169 (28.3%)	65.00 (13.16)	48.61 (13.91)	<.001
Online support group	127 (21.2%)	66.17 (12.99)	49.76 (14.31)	<.001
School professional	291 (48.7%)	60.88 (13.30)	46.00 (14.02)	<.001
Physician	314 (52.5%)	57.73 (15.42)	48.28 (14.16)	<.001
Minister or faith healer	111 (18.6%)	61.75 (14.50)	51.30 (15.15)	<.001
Parenting classes	254 (42.5%)	61.38 (13.72)	47.24 (14.04)	<.001
Social supports	286 (47.8%)	59.83 (13.83)	47.21 (14.58)	<.001
Telepsychology	211 (35.3%)	63.83 (12.51)	47.47 (13.94)	<.001
**Service utilized**				
Psychological counseling	210 (35.1%)	64.41 (11.66)	47.20 (13.99)	<.001
Crisis hotline	33 (5.5%)	76.24 (10.25)	51.90 (14.75)	<.001
Psychiatric hospitalization	29 (4.8%)	74.83 (9.90)	52.14 (14.98)	<.001
Mentorship programs	88 (14.7%)	69.09 (12.23)	50.51 (14.40)	<.001
Online support group	77 (12.9%)	69.70 (11.64)	50.81 (14.56)	<.001
School professional	222 (37.1%)	63.47 (12.70)	47.20 (13.85)	<.001
Physician	258 (43.1%)	59.46 (14.39)	48.53 (14.75)	<.001
Minister or faith healer	66 (11%)	64.55 (13.85)	51.84 (15.19)	<.001
Parenting classes	96 (16.1%)	66.70 (13.72)	50.67 (14.54)	<.001
Social supports	244 (40.8%)	59.57 (13.58)	48.88 (15.35)	<.001
Telepsychology	117 (19.6%)	65.94 (12.99)	50.15 (14.54)	<.001

% for total by column.

*p*-values represent Kruskal-Wallis test.

All items were more likely to be endorsed if youths had elevated total problems (Chi-square test of independence; *p* < .001).

*F* = frequency, *M* = mean, *SD* = standard deviation.

### Perceived service need

#### Measurement structure.

**Fig 1:**
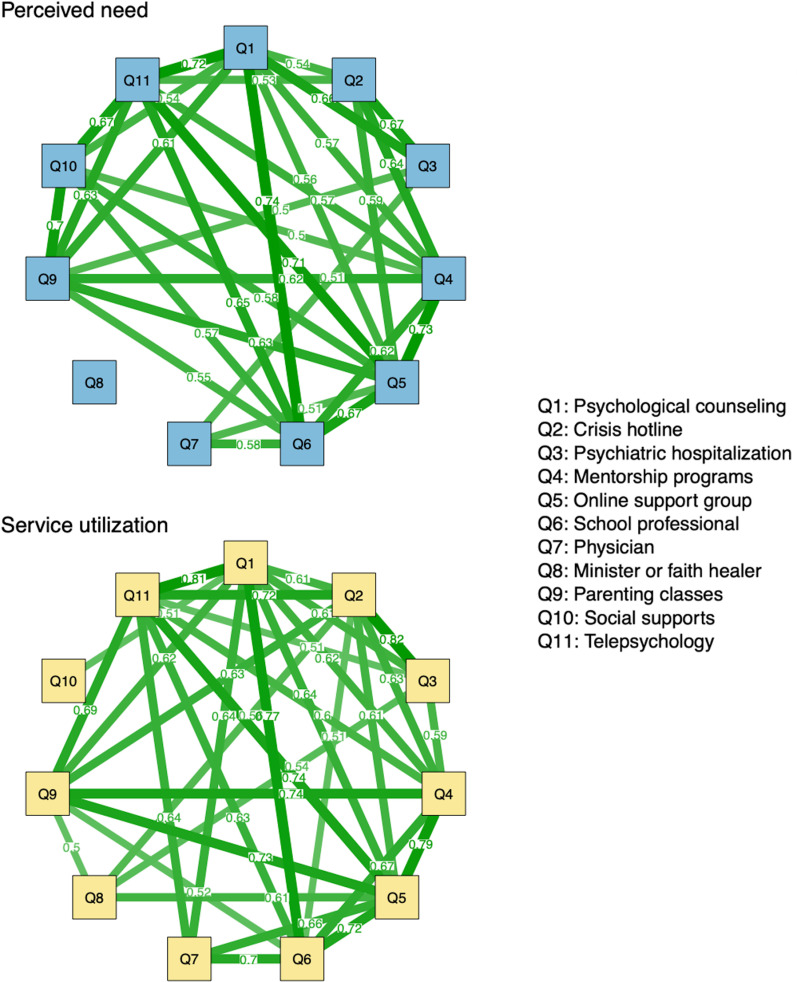
Tetrachoric correlations between Caregiver Support Services Questionnaire items.

Individual perceived need items differed in their frequency of endorsement with caregivers most commonly reporting needing youth supports such as physicians, social supports, school professionals, and psychological counseling (see Table 1). Caregivers reported needing help for their child from an average of 3.56 (*SD* = 2.96) different support services. While caregivers who reported needing any single support service for their child on the CSSQ were significantly more likely to report needing other sources of support, effects ranged from small (*r*_tet_ = .33) to large (*r*_tet_ = .74), which suggest that responses on these items are related but provide unique information. See Fig 1 for moderate to large tetrachoric correlations between CSSQ items.

Only moderate or greater correlations displayed to aid interpretability (≥.50).

**Fig 2:**
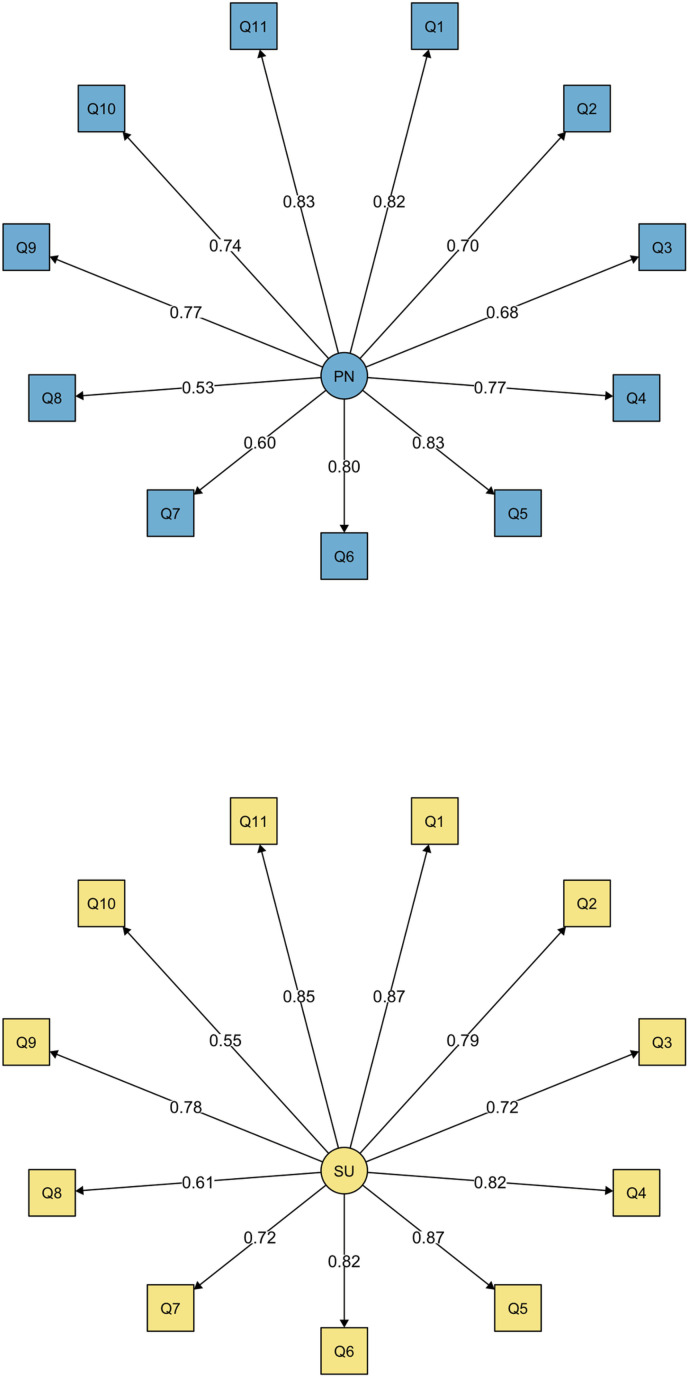
Confirmatory factor analysis of Caregiver Support Services Questionnaire scales.

A total cumulative scale was calculated for perceived need with measure items loaded onto a single factor. The cumulative perceived need model had good model fit across comparative indices according to their recommended cutoffs (RMSEA = .040 [90% confidence interval low = .028, high = .053; *p* = .893], TLI = .990, CFI = .992). See Fig 2 for the perceived need single factor model. Factor loadings for the culminative perceived need scale ranged from .53-.83. Loading the perceived need CSSQ items into a cumulative score resulted in an index with excellent internal consistency (α = .83).

#### Construct validity.

**Fig 3:**
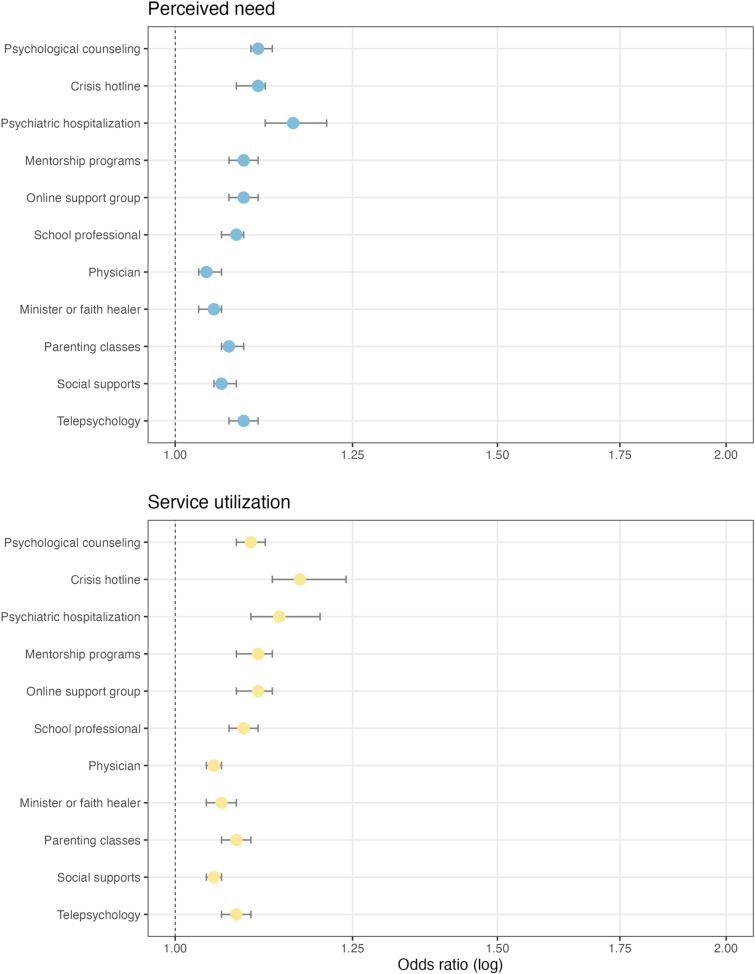
Caregiver Support Services Questionnaire items by emotional and behavioral problems. Variables whose confidence bands cross the 1.00 odds ratio line are not statistically significant at *p* < .05.

Perceived need items were related to youth problem severity as a cumulative score and individual items. Youth CBCL total problems T-scores (τ = .53, *p* < .001) had a moderate positive association with cumulative perceived need. At the individual item level, caregivers’ endorsement of perceived need items was generally associated with greater total youth problem severity relative to those who reported needing no services (*p* < . 001; See Table 1). Specifically, logistic regression analyses suggest that for each one unit increase in youth CBCL total problems T-scores, there was a 4.0-16.0% increase in the odds that caregivers would report needing a support service on CSSQ items (see Fig 3; S1 Table provides specific odds ratios, confidence intervals, and *p* values). However, the average youth CBCL total problems T-scores varied among caregivers that endorsed CSSQ perceived need items with some supports being associated with clinical (i.e., psychological counseling, crisis hotline, psychiatric hospitalization, mentorship program, online support group, telepsychology), borderline (i.e., school professional, minister or faith healer, parenting classes), or subclinical problems (i.e., physician, social supports).

### Service utilization

#### Measurement structure.

Individual service utilization items differed in their frequency of endorsement with caregivers most commonly reporting needing youth supports such as physicians, social supports, school professionals, psychological counseling, and parenting classes (see Table 1). Caregivers reported utilizing an average of 2.41 (*SD* = 2.58) for their youth. Cumulative service utilization had a significant positive association with responses on the perceived need scale (τ = .70, *p* < .001), which suggests that these constructs were strongly related but distinct. Caregiver reported use of support services on the CSSQ were intercorrelated with effects ranging from small (*r*_tet_ = .30) to large (*r*_tet_ = .81). This finding suggests that responses on CSSQ service use items were interrelated but largely provide unique information. See Fig 1 for moderate to large tetrachoric correlations between CSSQ items.

A total cumulative scale was calculated for service utilization with measure items loaded onto a single factor. The cumulative service utilization model had good model fit across comparative indices according to their recommended cutoffs (RMSEA = .042 [90% confidence interval low = .030, high = .054; *p* = .848], TLI = .990, CFI = .992). See Fig 2 for the service utilization single factor model. Factor loadings for the cumulative service use ranged from .55-.87. Summing service utilization CSSQ items into a cumulative score resulted in an index with excellent internal consistency (α = .83).

#### Construct validity.

Service utilization items were also related to youth problem severity as a cumulative score and individual items. Youth CBCL total problems T-scores (τ = .49, *p* < .001) had a moderate positive association with cumulative service utilization. At the individual item level, caregivers’ endorsement of service utilization items was generally associated with greater total youth problem severity (*p* < . 001; See Table 1). Logistic regression analyses suggest that for each one unit increase in youth CBCL total problems T-scores, there was a 5.0-18.0% increase in the odds that caregivers would report needing a support service on CSSQ items (see Fig 3; S1 Table provides specific odds ratios, confidence intervals, and *p* values). However, the average youth CBCL total problems T-scores varied among caregivers that endorsed CSSQ service utilization items with some supports being associated with clinical (i.e., psychological counseling, crisis hotline, psychiatric hospitalization, mentorship program, online support group, school professional, minister or faith healer, parenting classes, telepsychology) and subclinical problems (i.e., physician, social supports).

## Discussion

Research is needed to identify targets for reducing youth MHS disparities among Latinx families [1]. This gap in the literature may be in part explained by a lack of self-administered measures that can increase the feasibility of large-scale examinations of service utilization patterns among Latinx families. The present study sought to fill this gap through the creation and validation of the CSSQ, a self-administered questionnaire for assessing youth support service need/utilization among Latinx families. Our analyses facilitated by the CSSQ suggest that Latinx caregivers report needing and utilizing a wide range of youth support services. Correlations between CSSQ items within each scale had small to moderate effect, which suggest items were interrelated but provide unique information. Confirmatory factor analyses suggest that loading items in each CSSQ scale onto unidimensional cumulative factors fit the data well and had excellent internal consistency. These findings suggest that in addition to individual items that are face valid, the CSSQ can also be represented by cumulative perceived need and service utilization scales. The severity of youth emotional and behavioral problems was generally associated with increased odds of endorsing individual CSSQ items and cumulative scales, which provides support for the construct validity of the measure. These findings suggest that both individual CSSQ items and cumulative scores can be used to study caregiver MHS need and utilization in response to youth mental health problems.

Caregivers’ endorsement of individual CSSQ items varied by youth problem severity, which provides additional construct validity by highlighting perceptual thresholds for help-seeking that are consistent with theory. For example, the average youth problem severity differed among caregiver who perceived a need for services based on whether youth problems were in the clinical (i.e., psychological counseling, crisis hotline, psychiatric hospitalization, mentorship program, online support group, telepsychology), borderline (i.e., school professional, minister or faith healer, parenting classes), or subclinical range (i.e., physician, social supports). As caregiver perceived need and utilization of MHS is based on cultural interpretations of youth mental health problems (severity, perceived as related to mental health) and selection of appropriate means of intervention, this pattern may represent Latinx caregivers using different types of supports depending on the severity of their youths emotional or behavioral problems [10,11]. Additionally, crisis hotlines and psychiatric hospitalization had the highest average total problems scores for both perceived need and service utilization items. Both of these findings are consistent with caregivers seeking higher levels of care for their youth based on greater problem severity [16]. Further research is needed to understand the mechanisms contributing to these differences in CSSQ item responses based on youth problem severity among Latinx caregivers.

### Implications for research

The CSSQ is not intended to replace established semi-structured interviews that provide detailed information regarding youth MHS utilization patterns. Instead, the CSSQ should be viewed as an alternative that increases the feasibility of large-scale data collection when using in-person or online self-administered surveys. Similar to established interview-based measures [7–9], the CSSQ could be used at the item level to study Latinx caregiver-reported need/utilization of youth support services. Individual items from the CSSQ provide descriptive information regarding common, and modern, sources of support that Latinx caregivers perceived a need for and used to address youth mental health problems. Beyond descriptive statistics, individual CSSQ items can also be used as outcome variables in logistic regression models to help identify correlates associated with youth MHS need and utilization among Latinx families [4,12,35]. Dichotomous indicators of service need and utilization collected by the CSSQ can also help study supports networks that caregivers use to address their youths emotional and/or behavioral needs [13]. Cumulative CSSQ scores can be used in path analysis models to explore mechanisms underpinning help-seeking behaviors among Latinx caregivers [23].

While the CSSQ was developed for the Pathways to Latinx Mental Health study that focused on Latinx caregivers, this measure could be adapted to examine youth support service need/utilization within and/or between other racial/ethnic groups. As caregivers perceived need and utilization of support services are culturally bound [10], we encourage researchers to adapt the CSSQ to their target population to increase the relevance of this measure. Future research may also consider adding additional sources of support to the CSSQ that are not currently assessed by existing service utilization measures (e.g., mobile health applications, and self-guided online interventions; [28].]

### Implications for clinical practice

Beyond research, a measure of MHS need and utilization has potential clinical utility. Psychosocial interviews conducted prior to the start of psychotherapy often include questions regarding mental health and health service utilization history [36]. Administering the CSSQ to Latinx families prior to the start of psychotherapy with youths could help provide a broader picture of supports that are being leveraged, determine preferences for service formats (e.g., in person therapy versus telepsychology; parenting classes), and could identify unmet needs that could be addressed through case management. Clinicians can also select individual CSSQ item as screening questions as they are meaningful on their own and may not require the use of the entire scale to function adequately. As Latinx caregivers help-seeking behaviors may be informed by cultural values/beliefs regarding psychopathology [24,37] and healing practices [38], information from the CSSQ could also be used to guide discussion on cultural interpretations of the causes of youth mental problems and appropriate avenues for intervening (Cultural Formulation Interview; [36]), particularly among caregivers who report high degrees of cumulative perceived need and access few services. These conversations could assist clinicians in identifying and address culturally bound perceptual factors that may impact treatment adherence (e.g., disagreement regarding whether the youth’s problems are most appropriately addressed by formal psychological services; [38]).

### Limitations and future directions

The present study has several limitations. As the study used a cross-sectional design, temporal precedence between predictors and outcomes cannot be established. Thus, findings should be interpreted as correlations. The current measure is reductive in its approach to assessing MHS need/utilization to reduce the time burden of administering the measure and avoid issues with sparsity in the endorsement of items that can hamper statistical analyses. The CSSQ does not provide information on recency and frequency of service need/use. As a result, responses could range from a single occurrence to consistent use of supports within a one-year period. Further, information on whether participants lived in urban or rural settings that may have impacted their ability to access services was not collected, which may have impacted responses on the CSSQ.

Data collection for the Pathways to Latinx Mental Health study was completed nearly three months after the first coronavirus pandemic related shelter in place order (i.e., mid-March 2020; [4]). As a result, caregivers reported need for and use of youth MHS varied as a function of local shelter in place orders (transferring from in person services to telepsychology; [35]). While demand for youth telepsychology remains, future research should examine post-coronavirus support service utilization patterns among Latinx families [3]. Additionally, the CSSQ may be a useful tool for examining support service patterns resulting from anti-immigrant rhetoric and policy actions, which could help inform/support advocacy efforts [19].

Several limitations of the study associated with the use of Qualtrics panels should be noted. Qualtrics only provided responses from participants that had complete and high-quality data. As a result, it was not possible to examine patterns of missingness and imputing missing values. This procedure may have introduced potential biases as individuals who were uncomfortable reporting on their child’s mental health problems and/or use of MHS may have been more likely to prematurely discontinue participating in the survey. Future research should confirm our findings using complete data or a recruitment method that allows for the imputation of missing values. The present study utilized a non-clinical sample, which may have contributed to the low reported need/use of psychiatric hospitalization and crisis hotlines.

The first phase of the Pathways to Latinx Mental Health study required participants to complete the survey in English as part of the inclusion criteria due to feasibility issues with recruiting sufficient Spanish-speakers through an online survey panel. The focus on Latinx caregivers who could complete the survey in English may have resulted in specific acculturation patterns. Acculturation level is known to impact youth service utilization patterns among Latinx caregivers [25]. Further, Latinx caregivers who primarily communicate in Spanish may experience more language-based barriers to care (e.g., discrimination related to language/accent; [39]). Thus, using a survey panel may have contributed to MHS utilization patterns that may differ from Latinx subgroups that have greater difficulties in accessing care for their youths. Future research must translate and examine the psychometric properties of the CSSQ in Spanish and include caregivers with a wider range of acculturation levels. However, it should be noted that the present sample was largely bicultural (71.5% were first or second-generation; 45.3% equally preferred English and Spanish) and that the current measure presents a significant first step toward developing instruments that can help understand mental health disparities within an understudied population.

### Conclusions

The CSSQ represents a self-administered measure for gathering information on youth support service need and use from Latinx caregivers. Our findings suggest that the CSSQ may be used as cumulative scores and individual indicators to examine caregiver reported support service need and utilization. The CSSQ is a valid tool for measuring support service need and utilization patterns associated with youth mental health problems that could help facilitate research seeking to end disparities in MHS access among Latinx children. The development of psychological measures is an iterative process. The present study represents a step forward in the development of the CSSQ. An important future direction will be translating and validating the CSSQ in Spanish. Further adaptation could also improve the generalization of the measure to other racial/ethnic groups and include an assessment of emerging MHS (e.g., mobile health applications, and self-guided online programs).

## Supporting information

S1 Table Associations between perceived need and service use items and total youth mental health problems. (DOCX)

S1 Data Study data. (SAV)

S1 Text Caregiver Support Service Questionnaire (CSSQ). (DOCX)
