# Supplementary material for: Apoyando a la juventud [supporting the youth]: Latinx caregivers’ assessment of youth mental health service need and utilization on the Caregiver Support Services Questionnaire
Source: PLOS Ment Health. 2025 Jun 20;2(6):e0000345. doi: 10.1371/journal.pmen.0000345 (PMC12798168; doi:10.1371/journal.pmen.0000345)
Supplement: S1 Table — (DOCX) [file pmen.0000345.s001.docx]

| **S1 Table. Associations between perceived need and service use items and total youth mental health problems.** | | | | |
| --- | --- | --- | --- | --- |
| **Supports** | **OR** | **2.50%** | **97.50%** | ***p*** |
| **Perceived need** |  |  |  |  |
| Psychological counseling | 1.11 | 1.10 | 1.13 | <.001 |
| Crisis hotline | 1.11 | 1.08 | 1.14 | <.001 |
| Psychiatric hospitalization | 1.16 | 1.12 | 1.21 | <.001 |
| Mentorship programs | 1.09 | 1.07 | 1.11 | <.001 |
| Online support group | 1.09 | 1.07 | 1.11 | <.001 |
| School professional | 1.08 | 1.06 | 1.09 | <.001 |
| Physician | 1.04 | 1.03 | 1.06 | <.001 |
| Minister or faith healer | 1.05 | 1.03 | 1.06 | <.001 |
| Parenting classes | 1.07 | 1.06 | 1.09 | <.001 |
| Social supports | 1.06 | 1.05 | 1.08 | <.001 |
| Telepsychology | 1.09 | 1.07 | 1.11 | <.001 |
| **Service utilization** |  |  |  |  |
| Psychological counseling | 1.10 | 1.08 | 1.12 | <.001 |
| Crisis hotline | 1.18 | 1.13 | 1.24 | <.001 |
| Psychiatric hospitalization | 1.14 | 1.10 | 1.20 | <.001 |
| Mentorship programs | 1.11 | 1.08 | 1.13 | <.001 |
| Online support group | 1.11 | 1.08 | 1.13 | <.001 |
| School professional | 1.09 | 1.07 | 1.11 | <.001 |
| Physician | 1.05 | 1.04 | 1.06 | <.001 |
| Minister or faith healer | 1.06 | 1.04 | 1.08 | <.001 |
| Parenting classes | 1.08 | 1.06 | 1.10 | <.001 |
| Social supports | 1.05 | 1.04 | 1.06 | <.001 |
| Telepsychology | 1.08 | 1.06 | 1.10 | <.001 |
| CBCL = Child Behavior Checklist.  OR = odds ratio. | | | | |
