# Supplementary material for: Apoyando a la juventud [supporting the youth]: Latinx caregivers’ assessment of youth mental health service need and utilization on the Caregiver Support Services Questionnaire
Source: PLOS Ment Health. 2025 Jun 20;2(6):e0000345. doi: 10.1371/journal.pmen.0000345 (PMC12798168; doi:10.1371/journal.pmen.0000345)
Supplement: S1 Text — (DOCX) [file pmen.0000345.s003.docx]

**Caregiver Support Service Questionnaire (CSSQ)**

**In the past year**, did you think **your child** **needed or would have benefited** from any of the following support services?

|  | Yes | No |
| --- | --- | --- |
| Psychological counseling or therapy? |  |  |
| A crisis hotline? |  |  |
| Admission to a psychiatric ward or unit of a hospital? |  |  |
| A mentorship program (e.g. Big Brother, Big Sister)? |  |  |
| An internet support group? |  |  |
| A professional at school (e.g., guidance counselor, school psychologist, school social worker)? |  |  |
| A family doctor or any other medical doctor? |  |  |
| A minister (priest, rabbi, etc.) or faith healer? |  |  |
| Other (please specify) |  |  |

**In the last year**, did you think **you** **needed or would have benefited** from a parenting class to help raise your child?

- Yes
- No

**In the last year**, did you think **you** **needed or would have benefited** from help from social supports (e.g., family or friends) to raise your child?

- Yes
- No

**In the last year**, did you think **your child** **needed or would have benefited** from receiving psychological counseling over internet-based video conferencing?

- Yes
- No

**In the last year**, did **your child** **receive** help from any of the following support services?

|  | Yes | No |
| --- | --- | --- |
| Psychological counseling or therapy? |  |  |
| A crisis hotline? |  |  |
| Admission to a psychiatric ward or unit of a hospital? |  |  |
| A mentorship program (e.g. Big Brother, Big Sister)? |  |  |
| An internet support group? |  |  |
| A professional at school (e.g., guidance counselor, school psychologist, school social worker)? |  |  |
| A family doctor or any other medical doctor? |  |  |
| A minister (priest, rabbi, etc.) or faith healer? |  |  |
| Other (please specify) |  |  |

**In the last year**, did **you** **attend** a parenting class to help raise your child?

- Yes
- No

**In the last year**, did **you receive** help from social supports (e.g., family or friends) to raise your child

- Yes
- No

**In the last year**, did **your child** receive psychological counseling over internet-based video conferencing?

- Yes
- No
